# Arteriovenous Passage Times and Visual Field Progression in Normal Tension Glaucoma

**DOI:** 10.1155/2013/726912

**Published:** 2013-10-24

**Authors:** Eva Charlotte Koch, Kay Oliver Arend, Marion Bienert, Andreas Remky, Niklas Plange

**Affiliations:** ^1^Deptartment of Ophthalmology, RWTH Aachen University, Pauwelsstraße 30, 52057 Aachen, Germany; ^2^Eye Center Alsdorf, Cäcilienstraße 9, 52477 Alsdorf, Germany; ^3^Krankenhaus Barmherzige Brueder, Augen-Beleg-Klinik, 93049 Regensburg, Germany

## Abstract

*Purpose*. Fluorescein angiographic studies revealed prolonged arteriovenous passage (AVP) times and increased fluorescein filling defects in normal tension glaucoma (NTG) compared to healthy controls. The purpose of this study was to correlate baseline AVP and fluorescein filling defects with visual field progression in patients with NTG. *Patients and Methods*. Patients with a follow-up period of at least 3 years and at least 4 visual field examinations were included in this retrospective study. Fluorescein angiography was performed at baseline using a confocal scanning laser ophthalmoscope (SLO, Rodenstock Instr.); fluorescein filling defects and AVP were measured by digital image analysis and dye dilution curves (25 Hz). Visual field progression was evaluated using regression analysis of the MD (Humphrey-Zeiss, SITA-24-2, MD progression per year (dB/year)). 72 patients with NTG were included, 44 patients in study 1 (fluorescein filling defects) and 28 patients in study 2 (AVP). *Results*. In study 1 (mean follow-up 6.6 ± 1.9 years, 10 ± 5 visual field tests), MD progression per year (−0.51 ± 0.59 dB/year) was significantly correlated to the age (*P* = 0.04, *r* = -0.29) but not to fluorescein filling defects, IOP, or MD at baseline. In study 2 (mean follow-up 6.6 ± 2.2 years, 10 ± 5 visual field tests), MD progression per year (−0.45 ± 0.51 dB/year) was significantly correlated to AVP (*P* = 0.03, *r* = 0.39) but not to age, IOP, or MD at baseline. *Conclusion*. Longer AVP times at baseline are correlated to visual field progression in NTG. Impaired retinal blood flow seems to be an important factor for glaucoma progression.

## 1. Introduction

Glaucoma is a chronic progressive disease and one of the most frequent causes leading to blindness in the Western world, characterized by damage of the optic nerve and visual field loss. The pathogenic concepts of glaucoma may be divided into a mechanical, pressure-related, and a vascular approach. In addition to the individually increased intraocular pressure (IOP), other risk factors for the development of glaucoma like increased age, thinner central corneal thickness, and morphological changes like exfoliation syndrome, peripapillary atrophy, greater cup-to-disk ratio, and others have been identified [1–3].

For progression of glaucoma the following risk factors have been explored: Increased and unsteady IOP [1–4], older age [1–5], higher visual field mean deviation (MD), bilateral glaucoma [4], thinner central corneal thickness, and morphological changes like exfoliation syndrome, peripapillary atrophy, and disc haemorrhages [1–4, 6]. Additionally, vasospastic syndromes like hypotension and migraine [2] and diabetes mellitus [2, 3] have been described to influence progression. 

In addition, impaired ocular blood flow seems to play an important role in the pathogenesis of glaucoma [7–10]. Using fluorescein angiography, arteriovenous passage (AVP) times and fluorescein filling defects can be determined. Previous studies have shown that the AVP time is prolonged in primary open-angle glaucoma (POAG) and normal tension glaucoma (NTG) [7, 11]. Patients with asymmetric glaucomatous visual field loss have a significantly prolonged AVP time in the hemifield with the larger visual field defect [12]. Fluorescein filling defects of the optic nerve head represent areas of capillary nonperfusion and have been described in glaucomatous optic neuropathy [9]. The number, extend, and topography of the fluorescein filling defects agree with the visual field loss [10, 13, 14]. Patients with glaucomatous defects show absolute filling defects more often and with greater number compared to patients with ocular hypertension or healthy controls [10, 14].

The present study investigates the association of visual field progression in NTG with altered ocular blood flow, that is, AVP and fluorescein filling defects. Additionally, the influence of known risk factors (i.e., age, IOP, and the extent of the visual field damage) on the progression was analyzed in a multivariate approach.

## 2. Methods

The present study is an extension of previous cross-sectional studies on fluorescein angiography in normal tension glaucoma [10–12]. All patients with NTG had a detailed ophthalmological examination, visual field testing, and fluorescein angiography using a scanning laser ophthalmoscope (SLO Rodenstock Instr., Germany) at baseline. AVP times, fluorescein filling defects, IOP, age and visual field MD at baseline were used for analysis and correlated to the visual field progression in a multivariate approach. These baseline data were correlated to the visual field development in the following at least 3 years.

In this retrospective clinical study, AVP times and fluorescein filling defects were analyzed in two study groups (study 1: fluorescein filling defects, study 2: AVP). No overlap existed in these two study groups. Adherence to the Declaration of Helsinki for research involving human subjects is confirmed. Informed consent was obtained from all subjects. Fluorescein filling defects of the optic nerve head and retinal arteriovenous passage time were measured offline using digital imaging analysis (Matrox Inspector, Matrox Inc., QC, Canada) in a masked manner. The methods are described in detail elsewhere [7, 15]. The video fluorescein angiography was performed in a standardized manner. The 40-degree observation mode was used with the optic nerve head centred. To start the angiography, 10% sodium-fluorescein dye (2.5 cc) was injected into an antecubital vein. The image acquisition parameters (video gain and laser intensity) were kept constant until the maximum intensity levels in the retinal venules were passed over to avoid artefacts. Dynamic sequences were acquired with a frequency of 25 images per second. The retinal AVP time was determined using dye dilution curve analysis. The intensity level for each image was determined at a fixed region of interest (ROI) located on the arterioles respective venules. The extent of the ROI (circle) was chosen to correspond to the vessel diameter. Measurements were performed in the superotemporal and inferotemporal arterioles and venules. The mean retinal AVP time was used for analysis [7]. 

Images of the early phase (<3 minutes) were digitized visualizing the capillaries of the optic nerve head. During the angiogram, the focus was changed from the neuroretinal rim to the bottom of the cup to avoid artefacts. The extent of absolute fluorescein filling defects was measured in relation to the area of the optic nerve head (percentage of the optic disc). Absolute filling defects of the optic nerve head are defined as areas of persisting hypofluorescence during the entire angiogram [10, 14].

Visual field examinations were performed with the Humphrey Field Analyzer (Model 750, Humphrey-Zeiss, San Leandro, CA, USA, SITA program 24-2). MD was used for analysis. Visual field progression of each patient with NTG was analyzed using the MD progression per year method [16, 17]. A regression analysis of all available MD values during follow-up was performed. The MD progression per year was then calculated. This method allows the comparison of different periods of follow-up and different numbers of performed visual field examinations. The precondition to analyze the visual field tests was to fulfill the criteria of reliability, that is, false positive fault ≤ 20% and false negative fault ≤ 30%. IOP was measured using Goldmann applanation tonometry.

## 3. Patients

Patients with NTG and a follow-up period of at least three years and at least four visual field examinations were included in this retrospective longitudinal study.

Patients with NTG had a glaucomatous excavation of the optic disc and a glaucomatous visual field defect as defined by the European Glaucoma Society [17]. The diagnostic criteria for glaucomatous visual field loss are as follows. Field loss was considered significant when (a) glaucoma hemifield test was abnormal, (b) 3 points were confirmed with *P* < 0.05 probability of being normal (one of which should have *P* < 0.01), not contiguous with the blind spot, or (c) corrected pattern standard deviation (CPSD) was abnormal with *P* < 0.05. All parameters were confirmed on two consecutive visual fields performed with Humphrey Field Analyzer. All patients with glaucomatous visual field loss underwent diurnal curves of IOP measurements (Goldmann applanation tonometry) at 8.00 h, 12.00 h, 16.00 h, 20.00 h, and 24.00 h without any topical or systemic IOP-lowering medication. In patients with NTG, diagnosis was confirmed by readings of IOP never above 21 mmHg.

Between 1996 and 2003, 89 patients with NTG received a fluorescein angiography using scanning laser ophthalmoscope, in line with diurnal curves of IOP measurements and maximal clinical diagnostics for glaucoma patients. 72 patients fulfilled inclusion criteria, 44 patients for study 1 (fluorescein filling defects) and 28 patients for study 2 (AVP). 

The exclusion criteria for both studies were the presence of diabetes mellitus, allergy to sodium fluorescein, secondary glaucoma, PEX, other ocular (e.g., arterial or venous occlusion) diseases affecting ocular circulation, visual function, and diseases of the optic pathway.

In study 1 (fluorescein filling defects), 25 patients received no topical treatment at baseline. 19 patients were on local IOP-lowering medications (carbonic anhydrase inhibitors, ß-blockers, brimonidine, prostaglandins, or combinations). In study 2 (AVP), 18 patients had no topical treatment at the beginning of the study. 10 patients received local IOP-lowering medications (carbonic anhydrase inhibitors, ß-blockers, brimonidine, prostaglandins, or combinations).

## 4. Statistical Analysis

Correlations between MD progression per year and fluorescein filling defects and AVP were tested using a multiple regression analysis (Med Calc, Version 12.3.0, Belgium). Age, MD, and baseline IOP were included in the model as additional factors in both studies. In all analyses, *P* < 0.05 was regarded as statistically significant.

## 5. Results

**Figure 1:**
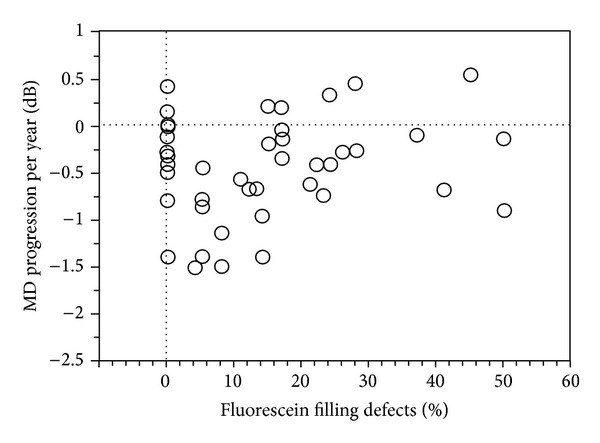
Bivariate plot of fluorescein filling defects (*P* = 0.37, *r* = −0.14) as a function of MD progression per year in patients with NTG.

In study 1 (fluorescein filling defects), mean follow-up period was 6.6 ± 1.9 years (range 3.2–11) and each patient underwent on average 10 ± 5 visual field tests (range 4–21). MD progression per year was significantly correlated to age (*P* = 0.04, *r* = −0.29), but there was no significant correlation between MD progression per year and fluorescein filling defects (Figure 1), MD at baseline, and baseline IOP. 

**Figure 2:**
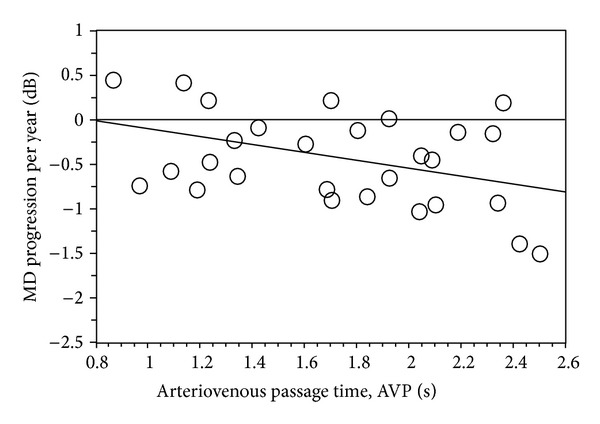
Bivariate plot of AVP (*P* = 0.03, *r* = −0.39) as a function of MD progression per year in patients with NTG.

In study 2 (AVP), mean follow-up period was 6.6 ± 2.2 years (range 3.2–11) and each patient underwent on average 10 ± 5 visual field tests (range 4–21). MD progression per year was significantly correlated to AVP time (*P* = 0.03, *r* = −0.39, Figure 2). But no significant correlation was found between MD Progression per year and age, MD at baseline, and baseline IOP.

Clinical data and results of the multiple regression analysis in study 1 and 2 are presented in Tables 1 and 2.

## 6. Discussion

There are several techniques to observe, measure, and interpret ocular blood flow [18]. However, each technique shows its limitations with respect to the interpretation concerning ocular blood flow. The aim of this study was to investigate an association of fluorescein angiography-based blood flow parameters (i.e., fluorescein filling defects and AVP) with visual field progression in a long-term follow-up period. To date, only few studies have been published, concerning the effect of impaired ocular blood flow on future glaucoma progression.

In 2003, Satilmis et al. showed that the progression rate of glaucomatous visual field damage correlates with retrobulbar end-diastolic velocity of the central retinal artery in a color Doppler imaging study. In a retrospective study, 20 patients with primary open-angle glaucoma were included, with a mean follow-up time of 4.3 years. Glaucoma progression was evaluated calculating the angle of the slope of a regression line of the visual field index mean defect over time compared to a horizontal line [19]. Janulevičiene et al. examined 30 patients with POAG in a prospective treatment study over a follow-up period of 18 months. Glaucoma progression was identified by standard automated perimetry and optic disc changes. Patients with glaucomatous progression had a higher nerve fiber index, lower systolic blood pressure, ocular and diastolic perfusion pressure, higher ophthalmic and central retinal artery vascular resistance, and lower pulse volume [20]. 

Other retrospective color Doppler imaging studies have been published in patients with POAG and NTG using different approaches in determining visual field progression. However, different perfusion parameters of the ophthalmic, central retinal and ciliary arteries were found to be associated with progression. [20–27].

Yamazaki and Drance included 31 patients with NTG and 28 patients with POAG in a 5-year follow-up study. In progressive NTG patients, lower blood flow velocities and higher resistive indices in the short posterior ciliary arteries and central retinal artery compared to patients with a stable visual field were found [22]. Zeitz et al. observed 114 patients with NTG and 40 healthy volunteers during a mean follow-up of less than one year. An increase in the cup-to-disc ratio of the optic nerve head in combination with a decrease of the mean deviation in visual field testing was defined as glaucoma progression. Patients with progressive disease showed a decreased peak-systolic velocity and end-diastolic velocity in the short posterior ciliary artery and a decreased peak-systolic velocity in the central retinal artery compared to patients with stable glaucoma and healthy volunteers [23] Interestingly, Yamazaki and Drance and Zeitz published the only progression studies with special focus on NTG patients investigating the impact of altered ocular blood flow on progression.

Another method for evaluating ocular blood flow is laser Doppler flowmetry. Zink et al. examined 23 patients with glaucoma with a mean follow-up of 33 months and 2 to 11 visual fields. Progression of glaucoma was evaluated by the slope of the corrected pattern standard deviation values over time. Optic nerve blood volume in the inferior temporal neuroretinal rim of the optic nerve was associated with CPSD progression [28].

In the present study, no correlation between fluorescein filling defects and glaucoma progression was found. This is not surprising, as fluorescein filling defects are strongly associated with current visual field damage and optic nerve head damage in glaucoma [9, 10, 13, 14, 29]. The capillary dropout as detected by fluorescein angiography seems to reflect the vascular damage in glaucoma at distinct time points but does not refer to the future trend of visual field defects. This is in accordance with previous fluorescein angiographic progression studies. Talusan et al. examined 17 patients with ocular hypertension, 14 patients with primary open-angle glaucoma, and 5 patients with normal tension glaucoma in a retrospective study during a follow-up period of 4 to 63 months using fluorescein angiography. Fluorescein filling defects of the optic disc were evaluated in at least two examinations during follow-up. New visual field defects and increased disc cupping were associated with new absolute filling defects [30]. Tuulonen et al. examined 24 patients with ocular hypertension in a retrospective study. During a mean follow-up time of 3.9 years two optic disc fluorescein angiograms were obtained. The results illustrated a correlation between the increase of fluorescein filling defects and lower filling rates of retinal veins with glaucomatous progression [31]. That is, the extent of the filling defects was not associated with future progression, but the enlargement of the filling defects was linked to further visual field loss.

In contrast, a significant correlation between AVP and glaucoma progression in NTG was apparent in this multiple regression analysis. Several studies illustrated that patients with glaucoma show prolonged AVP [7, 11]. However, a considerable overlap of AVP has been found, reducing the discriminative power of this retinal perfusion parameter to distinguish patients with NTG from healthy subjects at current time points. The association of prolonged AVP with the risk of future progression in NTG patients might be an explanation for this. Circulatory abnormalities, as detected by longer AVP, may play a significant role in the progression of glaucoma.

In a previous study, AVP times correlated with the end-diastolic velocity of the central retinal artery [32]. This association emphasizes the findings of Satilmis et al., 2003, who found correlation of the end-diastolic velocity in the central retinal artery and the progression of glaucoma. Interestingly, we found no significant correlation for baseline IOP and glaucoma progression in both studies, possibly because of the treatment with topical medication. In addition, in this study, IOP over time and IOP fluctuations were not analyzed. This phenomenon was also observed in the study of Satilmis et al., 2003. Again, this stresses the importance and influence of ocular blood flow in NTG pathogenesis. The influence of age on glaucoma progression is in part confirmed in this study with a correlation of age and MD progression per year in study 1 (fluorescein filling defects).

A limiting factor of this retrospective study could be the possible influence of other systemic vascular diseases, topical and systemic medications possibly affecting ocular blood flow. A large controlled prospective study would be appropriate to investigate such confounding factors. 

In conclusion, these results demonstrate that the decreased ocular blood flow as shown by longer AVP times may play an important role in the pathogenesis and progression in NTG. The impact of the vascular risk factors for the progression of glaucoma is again emphasized by this study. 

## Figures and Tables

**Table 1:** Clinical data (mean ± SD) of patients with NTG in study 1 (fluorescein filling defects, *n* = 44) and study 2 (AVP, *n* = 28) with corresponding fluorescein angiographic blood flow parameters.

	Study 1 (fluorescein filling defects)	Study 2 (AVP)
Age (years)	64 ± 10	62 ± 11
MD at baseline (dB)	−8.9 ± 7.8	−9.4 ± 7.6
IOP at baseline (mmHg)	15.3 ± 2.8	16.3 ± 2.7
Follow-up (years)	6.6 ± 1.9	6.6 ± 2.2
Visual field tests	10 ± 5	10 ± 5
MD progression per year (dB)	−0.51 ± 0.59	−0.45 ± 0.51
Fluorescein filling defects (%)	16 ± 18	NA
AVP (sec)	NA	1.7 ± 0.48

NA: not applicable.

**Table 2:** Multivariate correlation analysis of visual field progression (MD progression per year) in patients with NTG of study 1 (fluorescein filling defects) and study 2 (AVP) with fluorescein angiographic parameters and clinical data.

	*P* value	Correlation coefficient
Study 1		
Fluorescein filling defects	0.37	−0.14
Age	0.04	−0.29
MD	0.66	0.14
IOP	0.87	−0.004
Study 2		
AVP	0.03	−0.39
Age	0.24	−0.34
MD	0.06	0.25
IOP	0.83	−0.02
